# FAITH, SOCIAL SUPPORT, AND END-OF-LIFE CARE PREPARATION AMONG LGBTQ+ OLDER ADULTS

**DOI:** 10.1093/geroni/igad104.1363

**Published:** 2023-12-21

**Authors:** Nik Lampe, Harry Barbee, Nathaniel Tran, Tara McKay

**Affiliations:** University of South Florida, Tampa, Florida, United States; Johns Hopkins University, Baltimore, Maryland, United States; Vanderbilt University, Nashville, Tennessee, United States; Vanderbilt University, Nashville, Tennessee, United States

## Abstract

Faith communities can provide older adults support as they plan for end-of-life care, but how this support unfolds for lesbian, gay, bisexual, transgender, and queer (LGBTQ+) people is uncertain. Although many LGBTQ+ people experience marginalization within faith communities, they also report experiences of acceptance and affirmation. This mixed-methods study investigates how LGBTQ+ older adults’ involvement in faith communities shape their end-of-life care perceptions and preparation. First, we analyze panel data from the Vanderbilt University Social Networks, Aging, and Policy Study (N=1,256) to assess the relationship between faith community involvement and levels of social support. We find that 63% of older LGBTQ+ respondents identify with a religious affiliation, and 31% attend religious services several times a year or more. Attending religious services several times a year are more is associated with having a spouse in the household (11.4 vs 7.1%, p<.05) and reporting larger personal networks (11.7 vs 9.9 individuals; p<.001). Service attendance is also associated with having support when seeking advice about important decisions and having social support from friends. In the second portion of our study, we analyze data from in-depth interviews with 47 LGBTQ+ older adults to understand how faith communities influence preparation for end-of-life care. Many interviewees described their faith communities as guides that help them select reliable healthcare providers and formulate their end-of-life care plans. Overall, these findings reveal important ways that faith communities can foster social support and promote optimal end-of-life care among older LGBTQ+ adults.

